# Extra Supplement—The Nursing Mirror

**Published:** 1890-08-16

**Authors:** 


Being the Extra Nursing Supplement of "The Hospital" Newspaper.

All Contributions for this Supplement should be addressed to the Editor, The Hospital, 140, Strand, London, W.C., and should have the word "Nursing" plainly written in left-hand top corner of the envelope.

### En Passant.

**NEATLY PUT.**—"The sittings of the Lords' Committee on Hospitals have been occupied the greater part of this month by an inquiry as to the nursing arrangements at the London Hospital. An ex-chaplain and several late probationers have made complaints as to the treatment of the nurses, but the great grievance seemed to be that their services had not been appreciated by the Institution. The reason for this was easily understood, after accounts of their careers were given by the officials of the hospital. The evidence of the Matron, of Dr. Fenwick, and Mr. Treves was eminently satisfactory as to the nursing arrangements, and as far as the inquiry is now gone none of the accusations against the Hospital have been substantiated."—*Nursing Notes.*

**SHORT ITEMS.**—Mrs. Brewer, in this month's *Sunday at Home*, discourses on the workhouses and infirmaries of Cirencester and Gloucester.—Sir Charles Russell's sister is the Superior of a Catholic Nursing Order in Canada.—Miss Pringle has undertaken the starting of a Catholic Nursing Institution in the North of London.—Sister Hatcher, of St. Thomas's, has retired on a pension of £50.—Sister Rose Gertrude writes that she is getting up sports for the little leper boys; one consists in trying to catch a well-greased pig. One would have thought that a woman who pitied the lepers might also have some pity for a pig.—Miss Vincent received a hearty welcome home from Eastbourne, where she has been staying for her health. A bouquet was presented to her by the probationers.—The B.N.A. proposes to produce a quarterly journal.

**ASYLUM ATTENDANTS.**— All asylum attendants ought to read Dr. Frederick Needham's address on "Thirty Years of Lunacy Practice," in last week's *British Medical Journal.* Dr. Needham refers especially to the good results achieved in asylums from the better class of attendant; he sympathizes heartily with the difficulties of the work, and gives some hints which are valuable. "Their duties are delicate and difficult, their hours long, the calls upon their vigilance and patience incessant. They have at once to act as masters and servants, as companions and educators, and sick nurses." Further on Dr. Needham says: "We have endeavoured by lectures and other means to instruct attendants in the duties of their calling with some measure of success, but there is no special training institution where they can be regularly and systematically prepared for their important duties. This is a distinct need, which I hope will soon be supplied." A writer in the *Lancet* says: "Nurses and attendants are on duty from six a.m. to eight p.m. During this time their anxiety is constant, they are surrounded by the most harrowing pictures of humanity, and are, as a rule, bathed as it were by an atmosphere whose tendency it is to deprave those that breathe it morally, physically, and intellectually. Their periods of leave may be reckoned as follows: Fourteen days annually and forty-two hours out of each month. In other words, they are at their work for 4,434 hours every year out of a possible 5,110. In general hospitals nurses may come and go, and the patients will not suffer much. In asylums it is not so. The more a nurse knows the individual peculiarities of the patients under her care the better is she enabled to guard against accidents and casualties and attend to and supply their wants. Long service is to be desired, and should therefore be encouraged."

**PAY OR BE PAID?**—The controversy as to whether probationers should pay or be paid is still raging on the other side of the Atlantic, and strangely enough, the preponderance of opinion seems to be in favour of the paying probationer. However, one nurse writes to say that as several training schools send their probationers out private nursing in the second year, it certainly seems unfair that the probationer should pay the school at the same time she is earning substantial sums for the school. The question is one which is bound to come forward in England shortly.

**A WORD OF CAUTION.** — A lady superintendent desires to caution her fellow matrons, and makes the following suggestion:—"In view of a recent occurrence among my newly engaged nurses, may it not be feasible in some way to advertise or classify the intemperate nurse? She is generally about forty years old, has seasons of temperance, and holds good testimonials. She manages with these testimonials to get into a good home, and then in time she disgraces herself and the institution. I can name three of these nurses who have come under my personal observation." We have put the three names sent us on our "black list," and it is under our contemplation to circulate this list amongst hospital authorities, as the Cautionary Information Card of the Charity Organisation Society is circulated amongst the subscribers. Any suggestions from lady superintendents will be welcome.

**A STRIKE AT MELBOURNE.**—All the night nurses of the Melbourne Hospital have sent in their resignations, owing to the alleged ill-treatment of Nurse Smith, who died. The following letter has been sent to the Chairman: "Sir,—We, the undersigned night nurses, wish to state to you a few facts—firstly, concerning the night nurse's treatment of our poor companion, Nurse Smith; secondly, nurse's conduct and treatment of us all. Nurse Smith's illness was reported to sister at once, and she took no active means to even alleviate her pains. Nurse went on duty when she was quite distressed with pain. On the second night she could not hear us speaking to her, although quite loudly, and she had to go and take charge of a ward, and do injustice to her patients as well as to herself. Nurse being too ill to go to the dining-room, sister would not even allow her room companion to take her a cup of tea, so nurse lay in her bed without even a drink until you asked a day nurse to attend her. Not content with neglecting nurse, sister took on herself to abuse the poor girl, and insinuate she was not really suffering, and to have seen the effects of sister's visit on poor Nurse Smith would have been sufficient to convince anyone of the cruelty. For our own part, we might state a number of grievances, but all we will say is we find it impossible to work under sister after the treatment as to Nurse Smith. We are sorry to have to annoy you, as you have made so many efforts for our comfort and welfare, but feel sure you will give these statements every consideration and fully inquire into our cause,—Yours, etc." Here follow the signatures of all the night nurses. The nurses have refused to let the matron enquire into their grievances, and a sub-committee has been appointed. It is time the rod of authority was taken up in the Melbourne Hospital; either the nurses are right or wrong, whichever way it is summary punishment should fall on someone.

### Dr. Tarrant's Mistake.

*(Concluded from page* lxxxi.*)*

But in a few days, as hardly any of the students were able to see No. 20, the excitement died out. Meantime, No. 20 had been steadily progressing; he was able to speak, not well or distinctly, but intelligibly; he had regained the partial use of his hands. But he did not use his power of speech much, for he proved to be a sullen, taciturn, disagreeable man. One day, as he lay apparently asleep, two dressers standing by his bed fell into conversation about his case.

"It's odd," said the first, "that there should be nothing peculiar about his head, but there can be no doubt about it, Tarrant is right; the man is suffering from injury to the cerebellum. I wonder whether he'll get over it?"

"Can't say," replied the second, "but I don't think Tarrant would be sorry if he didn't, nor should I for the matter of that, though, of course, we must do our best for him. Tarrant tells me that a post-mortem examination of this case would enable him completely to revolutionize the present theories about the grey matter. It seems a pity that an encephalon, which would mark a new era in medical science, should be reserved for the use of one shady individual, instead of being devoted to the good of the human race."

They did not look at No. 20 as they moved away, or they might have observed a somewhat sardonic smile on his face. He had evidently heard their conversation, and attached some importance to it, for he seemed in much better spirits during the rest of the day. When Dr. Tarrant came round in the afternoon, he at once entered into conversation with him.

"I suppose you know how I got this injury, doctor?"

"I have formed an opinion from the position and direction of the wound, but I have no certain information."

"Attempted suicide," said No. 20, with an unpleasant chuckle.

"I hope, sir, that your marvellous escape from death has caused you to repent of your rash determination."

"Repent! You will excuse my amusement, I do not mean to be rude; but I put it to you, is it likely? When I was sound in life and limb my mental troubles were such that I determined to shoot myself. Now that I have added bodily pain to mental anguish, do you suppose I have changed my mind? We need not go into the cause. I may be a criminal or a bankrupt, a wrongdoer or an injured victim, but at any rate, I had amply sufficient reasons for wishing to get out of this world. Are a shattered skull and half an ounce of lead in my brain calculated to make life pleasanter to me? You are talking nonsense, doctor."

Dr. Tarrant made no reply. He would have liked to refer to the more serious side of the question, but, although in no ways sceptical, he had never given much consideration to religious matters, and consequently was rather at a loss.

The stranger, seeing his advantage, continued: "I understand that my case is a strange one, and likely to add to the resources of science. If I die in this hospital no one will claim me, and you will, I believe, have the right of dissecting me. Then why keep me alive, doctor? I want to die a painless death, for I have suffered enough already."

Here Dr. Tarrant, who was getting nervous and confused, broke off the interview; but day after day doctor and patient had long conversations together, of which the subjects are unknown, as the nurses and dressers were always sent away at No. 20's request. As a result, No. 20 got more and more jubilant, his harsh laugh continually rang through the ward, and the nurses and matron were constantly persecuted with his coarse jokes. Dr. Tarrant, on the other hand, grew moody and abstracted; he ceased to give lectures and to operate, and seemed to avoid the society of his fellows. One evening, about seven, the dresser, to whom I have before referred, noticed the two whispering together. Then Dr. Tarrant got up and walked down the ward. On a table stood a small stoppered bottle, apparently left there by one of the nurses. The doctor took it up as if by chance, but the dresser could see his hand shaking, and, walking up again to the bed of No. 20, said in a low trembling voice:—

"How careless to leave chloroform about; a third of this—a third of this," here his voice failed for a second, "if taken accidentally, would prove fatal."

He put the bottle down by No. 20's bedside, and hurried away, his face set in a ghastly stare. A few minutes afterwards the nurse passing by noticed that No. 20 looked rather pale, and his face was shiny, as if with perspiration, otherwise he seemed all right. Half an hour afterwards No. 20 was found dead, with his extremities already cold. He died with the self-satisfied smile of a man who thinks he has done something rather smart.

For the next two days Dr. Tarrant did not appear, but on the third he sent the following note to his favourite dresser:—

Dear Carey,—I shall do the post-mortem on No. 20 myself to-morrow morning, and shall be glad of your assistance.—Yours faithfully, Erasmus Tarrant.

The body, stripped stark naked, lay on the table in the post-mortem room, and Dr. Tarrant, taking in hand a small surgical saw, rapidly removed the top of the skull. Scarcely had he done this than he started back with a cry. "Good heavens, all this for nothing!" and sinking into a chair buried his face in his hands. He pulled himself together in a few minutes, and then mumbled, in a broken voice, "Carey, I am not very well to-day. Will you excuse me for an hour or two?" He walked slowly out of the room with a feeble, bewildered step, crossed the big quadrangle of the hospital, and passed out of the main gate. From that day to this St. Botolph's has never caught a glimpse of its most eminent surgeon. The same evening the following letter was posted to the governors of the hospital:—

Dear Sirs,—Having come to the conclusion that I am not competent to perform the duties of surgeon to St. Botolph's, I beg to place my resignation in your hands.—Your obedient servant, Erasmus Tarrant.

With this letter Dr. Tarrant passed entirely out of the English medical world. Not a soul has heard the vaguest report concerning him; no one knows whether he is alive or dead. Although the circumstances of the case were full of suspicion, yet there was no tangible evidence forthcoming, and no further steps were taken in the matter. The completion of the post-mortem showed the cause of Tarrant's discomforture and flight. The man's brain was perfectly normal; the inverted cerebellum was a mere fiction of the absent doctor's scientific imagination, a brilliant, plausible fiction, but absolutely without foundation in fact. What had happened was this: The bullet, proceeding from a revolver held in the right hand, had thrust the left temple diagonally, driving before it by its impact the pieces of bone so as to form a complete perforation of the skull. These pieces of bone were not driven in with sufficient force to inflict any perceptible injury to the brain.* The bullet itself, instead of proceeding on its course, was diverted outwards, and travelled right round the head between the tables of the skull, finally lodging in the base of the skull, where it exercised considerable pressure on the cerebellum, so setting up the symptoms which had misled Dr. Tarrant. It was also clearly established that the cause of death was chloroform.

*Mr. Savory, of St. Bartholomew's, stated in the course of a lecture, that a case had come under his notice in which a bullet entering at one ear had travelled right round the skull to the other, and there made its exit without injury to the brain; an even more extraordinary case than the one quoted above, as the bullet would have to turn a much sharper corner.

### Life and High Life at Johannesburg.

We have received the following interesting letter from Miss Rose Blennerhassett, enclosing a cutting from the *Digger's News*. The cutting we will give next week :—

The enclosed letter, published in the *Digger's News*, may be of some interest to your readers : I am afraid, however, we look more important and flourishing on paper than we are in reality. Nurses seem to have rushed to Johannesburg, speculating on a " boom of sickness," to use local slang. Our home is the original one, founded by the townspeople, and managed by a committee of ladies and a finance committee of men. The other two homes are private speculations, so that we feel we have the greatest claim on the town. Besides the three nursing homes, all trembling on the verge of smash, and many private nurses, there is a hospital at the gaol, into which the deserving poor are admitted (men only) on condition that they remain locked up till discharged. There is also a fine hospital built on a hill overlooking the town, which is to be opened in a month or two. Behind it is the temporary hospital—a large bungalow, containing about sixty beds. Outside it, in sheds and tents, are the Kafir wards. The town very nearly obtained the nursing of the hospital for this home, but the Roman Catholics got it finally by one vote, and have established nuns of, I think, the Order of the Holy Family to nurse it.

Miss Sleeman and I went over it. We found it very clean, but we thought the heavy stuff dresses of the nuns, the wide sleeves and veil, looked most unworkmanlike. We were allowed to see the dressings done. The Sister took us to the bedside of a young fellow with a crushed heel. We were puzzled to see a large sheet of muslin, as large as half a jacket poultice, lying on the boy's bed, with great lumps of linseed strewn upon it. Could it be a poultice? Impossible. It looked quite cold. The Sister bandaged the foot with flannel, leaving the heel bare, she then plunged the whole foot into the linseed, tied it up in a bag, and put a newspaper under it. After this we thought it better to escape to the medical wards. Here we found some typhoids literally boiling in a small hot room. We asked if such a temperature wasn't bad for them. The Sister said it might be so, but that a throat case in the next ward required heat, and it was safer to heat both rooms for him, as they communicated. She also told us that the sisters did not sit up at night. Black boys do most of the night work. The male patients are not washed. The Kafirs lie on deal tables with a straw mat under them. We asked the Sister how she managed their backs. She said calmly, " They get sore." She told us that at one time the Kafirs had mattresses, but the committee had ordered them to be burnt, from which we inferred that they had got into a dirty state. All this will allow of people drawing their own conclusions as to the relative advantages of nuns and nurses. One pull the nuns have over us, they work for nothing, or nearly nothing. But I can't help feeling that it is a pity this hospital is not properly nursed and managed. The new hospital is large and splendidly situated, and in skilful hands might become a nursing centre for this part of Africa, after the fashion of Kimberley Hospital, where Sister Henrietta does such capital work.

As regards ourselves. Like most people whose finances are becoming more and more hopeless, we have become more comfortable. Miss Mollett has struck against cleaning and cooking, and got a white woman and a black boy called Sixpence, and between them we do fairly well. I mention this, because amusing letters from some of the nurses' friends followed the publication of my scribblings. Some sent recipes for cooking various dainty dishes, and offers of fish sauces, etc., by the next steamer ! I appear to have said we had had " fried fish " (instead of beef), but, alas, there is no such thing as a fish to be had in Johannesburg. Now and then, at rare intervals, a flabby, white fresh-water fish, partially salted, and fuller of bones than a herring, is hawked about, and bought by the unwary.

The ball given for the Nurses' Homes was a great success, over £300 being clear profit. Unfortunately, almost all of it had to go towards paying off a portion of the old debts. The ball itself was a curious spectacle. It would be perhaps indiscreet to describe many of the strange incidents which took place in a hall containing several hundred people, of whom a large proportion were very tipsy, or I should say " squiffy " before ten o'clock. The English clergyman played in the orchestra in Court dress !

Women of Society here consist chiefly of dressmakers and helps of all sorts. Nurses and doctors' wives are the *dessus du panier*. Barmaids are, I believe, exclusive and select, and keep to their own set. Some of them have very nice traps and horses, and they are said to be better paid and to enjoy life more than anyone else here.

This time last year doctors were attending an average of forty cases a day. This year there is little or no sickness. The town is no longer overcrowded ; numbers have left, and are leaving every day, and the Sanitary Board has a chance of keeping the place tolerably clean. The climate is good, the air simply splendid, and skies and sunsets of magical beauty. The country round about is a barren, dreary waste—not a tree to be seen. At least, I hear there is a tree and a waterfall and a baboon within an afternoon's drive. The baboon's appearances cannot, of course, be counted on, but we project going to try and make his acquaintance.

In a month or two we are told to expect the fleas. Every square inch of Johannesburg, indoor and out, is said to swarm with them during the summer ; and I can well believe it, for no one who hasn't been to Johannesburg and to the Transvaal generally, has the very faintest idea what *Dirt* means. Compared to this place, a Spanish village is a paradise of cleanliness.

Nurses who complain of monotony at home have little notion of the almost unbearable dulness of colonial life. When off duty there is absolutely nothing to do, nowhere to go, nothing to see ; cabs cost 7s. 6d. an hour—a price that forbids many drives to most of us ; there are no concerts, no artistic exhibitions or even shops ; a ridiculous theatre, the places very dear ; and wherever you go, and whoever you see, you are certain to hear nothing talked of but money—money—money.

### Everybody's Opinion.

[*Correspondence on all subjects is invited, but we cannot in any way be responsible for the opinions expressed by our correspondents. No communications can be entertained if the name and address of the correspondent is not given, or unless one side of the paper only be written on.*]

### MISS GEE'S HOME, MONTREAL.

" An English Woman " writes : A nurse asks in The Hospital last week for information respecting Miss Gee's home in Montreal, Canada. As I have only recently returned from Montreal, perhaps I can supply a little information. Miss Gee went to Montreal some four or five years ago from one of the London hospitals, and began as a private nurse entirely upon her own account. She has accumulated sufficient money to purchase the home in McGill College Avenue. It is a fairly-sized house in the west-end. She receives a few private patients, and supplies private nurses. I did not nurse for Miss Gee. I acted upon the advice of an old nurse who had been out there for some years, which was—get copies of your certificate and testimonials printed, and then get a list of the principal doctors out there, call upon them and leave a copy of certificate and testimonials and card with address, then, like Mr. Micawber, " wait " for something to turn up—

and you will not wait long. English nurses are wanted badly, as they have a very inferior class of nurses there. About this time of the year would be best to start, as typhoid is very prevalent, and a nurse can earn from two to four dollars per day. There are "directories" for nurses where you can register your name and qualifications upon paying a fee, and you get very good cases from these sometimes; the two I know are: 36, Cathcart Street; and Mrs. White's, Victoria Street. You can room and board at either of these, and, in fact, they are much like nurses' homes here, except that you take your own earnings. I would not discourage any nurse, but she will find Canada very different to old England; and I am not ashamed to confess that I was terribly home sick and the climate is very trying, too, as well as your having to rough it considerably.

### NURSING AT THE LONDON.

"One Who Values Her Training" writes: Having seen the article on "Nursing at the London Hospital, Before the Lords' Committee," in your issue of July 19th, I, as a former probationer, cannot help feeling indignant at the false charges brought against the matron. Having entered for my training the same year as Nurse Yatman, viz., 1883, I can speak from experience. Miss Lückes has always tried in every way to improve the tone of the hospital nurses as well as taken a deep interest in their comfort and welfare, and has so far succeeded in her efforts that their surroundings are far superior to what they were seven years ago, but being now matron of a hospital I have experienced how so many unsuitable women seek to take up the profession for their own selfish motives, not looking upon it as an honourable and self-denying calling, but looking for their own ease and comfort and to do as little work as ever they can, while seeking to be called by the name of nurse, of which title they are utterly unworthy. As to the "menial duties," any right-minded nurse should, no matter what her social standing may be, consider it a great privilege to be able to put her hand to any kind of work for the help of her fellow-creatures in suffering. If, as nurses, they have not learned to perform these so-called menial duties, how shall they, if they hope to become sisters or matrons, be able to teach others? Further, I despise the want of *esprit de corps* which would allow anyone calling herself a nurse to seek help from the chaplain, who, as an unqualified man, was hardly able to judge whether a nurse had sufficient done for her during her illness. Moreover, I should like to be informed whether it is the rule for the chaplain of the hospital to take the nurses' temperatures, which apparently was done, as given in evidence with reference to Nurse Powell?

### A HOME OF REST.

A "Lady Nurse" writes: "I will be much obliged if you will kindly insert the following in your much-read paper. I feel that I must write a few lines in answer to Miss Louisa Twining's letter, for if some one does not, people who know nothing about nurses may naturally think her view is a correct one. Can she be in earnest in saying 'At the present moment there is *trop de zêle* on behalf of nurses?' I am thankful to say that the general public do not think so, and she shows that she must know very little about the nurses of the present day when she states that they are mostly taken from the servant class. Most of our nurses of the present day are ladies, indeed it is very rare to find a nurse who belongs to the servant class, which Miss Twining somewhat contemptuously speaks of. Nurses of the present day have to be women of refinement and education. No one who belongs to the servants' class could pass the examinations and fill the responsible posts our nurses do nowadays. Miss Twining speaks of some years' experience in an infirmary. It is a bad argument to bring forward. We all know that until the last few years there was attendants in infirmaries but no trained nurses. A 'Home of Rest' for nurses is badly wanted, though, I am thankful to say, most of us have friends to go to; still sometimes we may be attacked with illness while at our work in a London hospital, miles away from our friends, who may live in the north or south, as the case may be. We are nursed well in our institutes until convalescent, and then the doctor will most probably advise a few days' rest before resuming work. It is not possible to go a long journey for a few days, to say nothing of the expense, which we can so ill afford. Then in these cases what a blessing a Home of Rest would be. It would be a real 'Home of Rest' to those who are weary and ill from working for and ministering to others. Surely all who know anything about nurses will encourage the idea instead of talking of *trop de zêle* on our behalf."—["A Lady Nurse" (?) seems to know so little about nursing that she does not recognise who Miss Louisa Twining is, nor by what supreme right she can speak about infirmaries and their nurses.—Ed.]

### Notes and Queries.

To Correspondents.—1. Questions may be written on post-cards. 2. Advertisements in disguise are inadmissible. 3. In answering a query please quote the number. 4. If a private answer is desired, a stamped addressed envelope must be forwarded.

### Queries.

(32) *Microscopes.*—Where can I obtain a good microscope cheap? Also what book would instruct me in mounting specimens?—*Sister Rachel.*

### Answers.

(26) *Nursing in Japan.*—The Doshisha Hospital in Japan is nursed by American trained nurses. The matron is Miss Richards. The St. Hilda Mission of London sends women to Japan, but more as missionaries than as nurses.—*Ego.*

*Hospital in Paris.*—If "Norma" had consulted back numbers she would have seen this query has been constantly answered. English nurses can obtain work in Paris at the Hertford Hospital, the Levick Institution, and at Miss Nelson's Home in the Rue des Acacias.

### Vacancies.

The following vacancies are announced:

**Nurses.**

Barnhill Poorhouse; £25.

Burton-on-Trent Institution; £25.

Bangor Institution.

Bournemouth Institution; £25.

Birmingham and Midland Hospital for Women; £23.

Blackheath and Richmond Institution.

Borough Fever Hospitals, Sheffield; £25.

Brixton Institution.

Bristol District Society.

Cheltenham Institution.

County of Cornwall Trained Nurses' Home; £25.

Fleming Memorial Children's Hospital, Newcastle.

Hanover Institute; £25.

Institution of Trained Nurses, Leicester; £25.

Kidderminster Infirmary.

Kent Institution; £30.

London and Brighton Association.

Levick Institution, Paris.

Leith Hospital; £25.

Ditto (assist.); £20.

Manchester Hospital for Consumption; £20.

Nurses' Home, Leeds.

Nurses' Home, Newcastle-on-Tyne.

Nurses' Home, Hull.

Nurses' Home, Bengeo, Herts; £30.

Nursing Institution, Wimpole St.

Oswestry and Ellesmere Cottage Hospital; £25.

Royal Hospital for Women and Children.

South-Eastern Fever Hospital; £30.

St. Helena Home.

St. Peter's Hospital for Stone; £22.

Surrey County Asylum; £18.

Sheffield Union; £20.

St. Saviour's Infirmary, S.E.; £20.

St. John's Institute, Southport; £25.

Swansea and South Wales Institute.

St. Olave's Union; £20.

Torquay Institution.

Tunbridge Wells Hospital; £25.

West Derby Union; £25.

Whitechapel Infirmary; £22.

Workhouse Infirmary Association.

Weston-Super-Mare Institute.

Whitchurch Cottage Hospital; £30.

**Matrons.**

Carlisle Fever Hospital; £50.
Stratford-on-Avon Hospital; £40.

**Probationers.**

Bristol Training Institution.
Children's Hospital, Birmingham.
Kilmarnock Infirmary.
North-West London Hospital; £8.
Sheffield Union; £10.
St. Helena Home.
Workhouse Infirmary Association.

**District Nurses.**

Mossley Sick Nursing Association.

Queen Victoria's Jubilee Institute (Scottish Branch).

### Amusements and Relaxation.

### SECOND QUARTERLY WORD COMPETITION.

Commenced July 5th, 1890, ends Sept. 27th, 1890.

Three prizes of 15s., 10s., 5s., will be given for the largest number of words derived from the words set for dissection.

Proper names, abbreviations, foreign words, words of less than four letters, and repetitions are barred; plurals, and past and present participles of verbs, are allowed. Nuttall's *Standard* dictionary only to be used.

N.B.—Word dissections must be sent in WEEKLY not later than the first post on Thursday to the *Prize Editor*, 140, Strand, W.C., arranged alphabetically, with correct total affixed.

The word for dissection for this, the SEVENTH week of the quarter, being

"MEDLARS."

| Names. | Aug. 7th. | Totals. | Names. | Aug. 7th. | Totals. |
|---|---|---|---|---|---|
| Dulcamara | 123 | 288 | Jenny Wren | 124 | 276 |
| Tinie | 129 | 299 | Rhoda | 105 | 242 |
| Henri | 115 | 275 | Annabel | — | 17 |
| Agamemnon | 135 | 303 | Spare Moments | — | 19 |
| Patience | 131 | 298 | Qu'appelle | 120 | 283 |
| Brisbane | 108 | 235 | Nurse | — | 29 |
| Nurse Hilda | 126 | 280 | A. B. C. | 113 | 182 |
| Lightowlers | 133 | 294 | Brickbat | 77 | 77 |

**Notice to Correspondents.**

*Brisbane: Result of Third Week* 40.

N.B.—Each paper must be signed by the author with his or her real name and address. A *nom de plume* may be added if the writer does not desire to be referred to by us by his real name. In the case of all prize-winners, however, the real name and address will be published.

Competitors can enter for *all* quarterly competitions, but no competitor may take more than *one first prize* during the year.

